# Diacylglycerol regulates acute hypoxic pulmonary vasoconstriction via TRPC6

**DOI:** 10.1186/1465-9921-12-20

**Published:** 2011-02-04

**Authors:** Beate Fuchs, Markus Rupp, Hossein A Ghofrani, Ralph T Schermuly, Werner Seeger, Friedrich Grimminger, Thomas Gudermann, Alexander Dietrich, Norbert Weissmann

**Affiliations:** 1 Excellence Cluster Cardio-Pulmonary System, University of Giessen Lung Center, Dept. of Internal Medicine II, Justus-Liebig-University Giessen, Giessen, Germany; 2 Max-Planck-Institute for Heart and Lung Research, Bad Nauheim, Germany; 3 Excellence Cluster Cardio-Pulmonary System, University of Giessen Lung Center, Dept. of Internal Medicine IV/V, Justus-Liebig-University Giessen, Giessen, Germany; 4 Walter-Straub-Institute for Pharmacology and Toxicology, Ludwig-Maximilians-University Munich, Munich, Germany

## Abstract

**Background:**

Hypoxic pulmonary vasoconstriction (HPV) is an essential mechanism of the lung that matches blood perfusion to alveolar ventilation to optimize gas exchange. Recently we have demonstrated that acute but not sustained HPV is critically dependent on the classical transient receptor potential 6 (TRPC6) channel. However, the mechanism of TRPC6 activation during acute HPV remains elusive. We hypothesize that a diacylglycerol (DAG)-dependent activation of TRPC6 regulates acute HPV.

**Methods:**

We investigated the effect of the DAG analog 1-oleoyl-2-acetyl-sn-glycerol (OAG) on normoxic vascular tone in isolated perfused and ventilated mouse lungs from TRPC6-deficient and wild-type mice. Moreover, the effects of OAG, the DAG kinase inhibitor R59949 and the phospholipase C inhibitor U73122 on the strength of HPV were investigated compared to those on non-hypoxia-induced vasoconstriction elicited by the thromboxane mimeticum U46619.

**Results:**

OAG increased normoxic vascular tone in lungs from wild-type mice, but not in lungs from TRPC6-deficient mice. Under conditions of repetitive hypoxic ventilation, OAG as well as R59949 dose-dependently attenuated the strength of acute HPV whereas U46619-induced vasoconstrictions were not reduced. Like OAG, R59949 mimicked HPV, since it induced a dose-dependent vasoconstriction during normoxic ventilation. In contrast, U73122, a blocker of DAG synthesis, inhibited acute HPV whereas U73343, the inactive form of U73122, had no effect on HPV.

**Conclusion:**

These findings support the conclusion that the TRPC6-dependency of acute HPV is induced via DAG.

## Introduction

Hypoxic pulmonary vasoconstriction (HPV) is an essential mechanism in the lung matching blood perfusion to alveolar ventilation, thus optimising gas exchange [1]. Despite decades of research, the signaling pathway underlying HPV has still not been fully resolved. An increase in intracellular calcium concentration ([Ca^2+^]_i_) is an essential component in this process, leading to the contraction of precapillary pulmonary arteries [2-4]. However, how [Ca^2+^]_i _is regulated in HPV is still a matter of debate [2,3,5,6]. In addition to L-type voltage-operated calcium channels (VOCC), non-selective transient receptor potential (TRP) channels have been suggested as important regulators of vascular tone in hypoxia [7-9]. In mammals, the family of TRP channels comprises 6 subfamilies, based on their sequence homology [10]. Among these, classical TRPC proteins are expressed in pulmonary arterial smooth muscle [4,5,9], specifically, in smooth muscle cells of distal pulmonary arteries [11], which are suggested to be O_2 _sensor and effector cells of acute HPV [12]. Focusing on these aspects, the transient receptor potential channel (TRPC) 6 has recently been identified to be essential for acute but not sustained HPV in mice [9]. In this regard it is important to mention that HPV has repeatedly been shown to consist of two phases. An acute phase occurring within several minutes and a sustained phase developing within more than 30 min of hypoxic ventilation [12-17]. TRPC6 belongs to the TRPC3/6/7 subfamily of TRP channels which can be activated by diacylglycerol (DAG) [18], independently of protein kinase C [19,20]. Hypoxia induces an accumulation of DAG in isolated pulmonary artery smooth muscle cells (PASMC) [9].

We therefore hypothesize that DAG contributes also to the regulation of acute HPV and that the DAG signaling pathway involves TRPC6 in intact lungs.

DAG synthesis results from activation of G-protein coupled receptors or receptor tyrosine kinases and subsequent activation of phospholipase C isoforms (PLCβ or PLCγ) leading to hydrolysis of phosphatidylinositol 4,5-bisphosphate (PIP_2_) [21]. The degradation of DAG is catalyzed by DAG kinases to phosphatidic acid (PA) [22].

## Materials and methods

### Animals

All animal experiments were approved by the local authorities. Adult C57/BL6 mice were obtained from Charles River Laboratories (Sulzfeld, Germany). TRPC6 deficient (TRPC6^-/-^) mice were generated as described previously [7]. Respective wild-type (WT) littermates from this colony were used as controls.

### Isolated lung perfusion and ventilation

The model of isolated, perfused mouse lungs has been described previously [23]. Briefly, lungs were excised under deep anesthesia, perfused with Krebs-Henseleit buffer (pH 7.37-7.40) in a recirculating system, and ventilated with a mixture of 21% O_2_, 5.3% CO_2 _and the balance N_2 _(normoxic ventilation). The pressure in the pulmonary artery and in the left atrium was measured by small diameter catheters.

### Induction of acute vasoconstriction

Repetitive hypoxic maneuvers of 10-minute duration interrupted by 15-min periods of normoxia were performed. The effects of the various pharmacological agents on pressure responses provoked by alveolar hypoxia (1% O_2_, 5.3% CO_2 _and the balance N_2_) were determined within such a sequence of repetitive hypoxic maneuvers. 1% O_2 _was chosen for hypoxic ventilation as this degree of hypoxia resulted in the most prominent HPV as described before also for other species [24]. Normoxic pulmonary arterial pressure was quantified directly before each hypoxic ventilation maneuver.

To evaluate the specific role of the agents applied in HPV intrinsic pathways, the effect on hypoxia-independent vasoconstriction was determined, using the thromboxane mimetic U46619 [25]. In these experiments, the ventilation remained normoxic, and the lungs were challenged with 4.5 nM U46619 (Paesel and Lorei, Duisburg, Germany; 5.7 mM stock solution in DMSO), applied as a bolus into the pulmonary arterial line. Such bolus applications were repeated every 25 min.

The experiments with TRPC6^-/- ^mice as well as their WT controls were performed during continuous normoxic ventilation.

### Application of the agents

After the second hypoxic maneuver, the DAG analog 1-oleoyl-2-acetyl-sn-glycerol (OAG), the DAG kinase inhibitor R59949, the PLC inhibitor U73122 or its inactive form (U73343) were applied into the recirculating perfusion medium 10 min prior to the next hypoxic or U46619 challenge with a stepwise increasing dose. For application stock solutions of OAG (50 mM; Sigma-Aldrich, Steinheim, Germany) and R59949 (100 mM; Calbiochem, Bad Soden, Germany) were prepared in DMSO (Merck, Darmstadt, Germany). U73122 and U73343 were dissolved in 96% Ethanol (Fischer, Saarbrücken, Germany) with a stock concentration of 1 mM.

Control experiments were performed with the application of the respective solvents alone.

### The effect of the DAG kinase inhibitor R59949 on sustained HPV

To investigate the effect of the DAG kinase inhibitor R59949 on sustained HPV lungs were ventilated for 120 min with a hypoxic gas mixture (1% O_2_, 5.3% CO_2 _and the balance N_2_) and R59949 was added to the perfusate 10 min prior to the onset of hypoxia. R59949 was applied in a concentration (10 μM) that inhibited acute HPV by approximately 50%. To characterize a possible sustained effect of R59949 on normoxic vascular tone this agent was applied in an analog schedule but during normoxic ventilation. Control experiments were carried out with the application of the solvent only.

### Analysis

The strength of acute HPV is given as the maximum increase in pulmonary arterial pressure (ΔPAP), referenced to the second hypoxic challenge (set at 100%). Changes of the normoxic pulmonary arterial pressure (ΔPAP) in these experiments were referenced to the normoxic PAP directly before the second hypoxic maneuver. The U46619 and normoxic experiments are displayed accordingly. For sustained HPV the increase in pulmonary arterial pressure is given. All values are expressed as means ± SEM (standard error of the mean). Statistical analysis was performed using ANOVA with the Student-Newman-Keuls *post hoc *test or Student's *t*-test with Welsh's correction as appropriate. A p-value < 0.05 was considered significant.

## Results

### OAG-induced activation of TRPC6 under normoxic conditions

**Figure 1:**
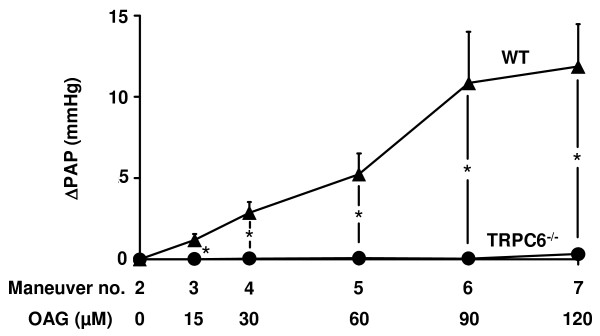
**No effect of 1-oleoyl-2-acetyl-sn-glycerol (OAG) on normoxic vascular tone in TRPC6^-/- ^mouse lungs**. The effect of OAG was investigated in isolated wild-type and TRPC6^-/- ^mouse lungs. The OAG-induced vasoconstriction is represented as the increase in pulmonary artery pressure (ΔPAP) during normoxic ventilation. OAG was applied in increasing doses every 25 min. Data are derived from n = 5 and n = 4 wild-type and TRPC6^-/-^mice, respectively. * Significant differences compared to wild-type mice (p < 0.05).

To decipher a possible role for DAG in TRPC6 activation, the effect of the membrane-permeable analog of DAG, OAG, on pulmonary artery pressure was investigated in TRPC6^-/- ^and WT mice. As illustrated in Figure 1, OAG increased PAP dose-dependently only in WT mice but not in TRPC6^-/- ^mice. PAP measured after the initial steady state period was 9.9 ± 0.1 mmHg (n = 9) and was not different between WT and TRPC6^-/- ^mice.

To address the role played by DAG in acute HPV, the effect of the DAG analog OAG as well as of inhibitors of both PLC and DAG kinase on HPV and normoxic vascular tone was assessed.

### The effect of OAG on acute hypoxia-induced vasoconstriction

**Figure 2:**
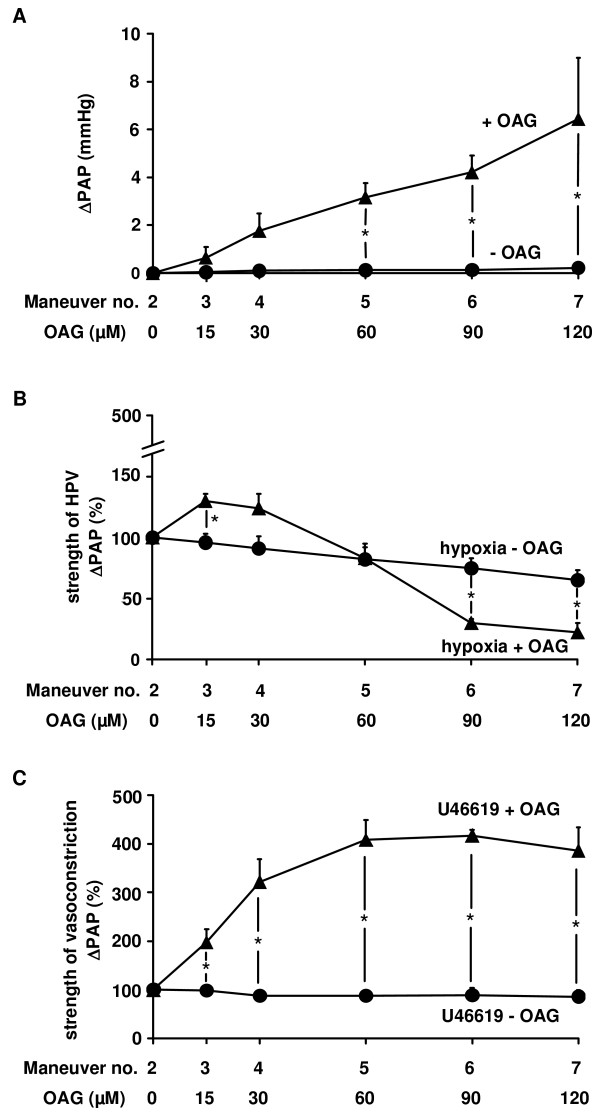
**1-oleoyl-2-acetyl-sn-glycerol (OAG) diminished HPV specifically**. The effect of OAG as well as its solvent on the normoxic vascular tone, the strength of HPV, and the strength of U46619-induced vasoconstriction was investigated in isolated wild-type mouse lungs. (A) Increase in normoxic pulmonary arterial pressure (ΔPAP), assessed directly before each hypoxic ventilation maneuver. (B) Strength of HPV referenced to the effect of the second hypoxic ventilation maneuver (= 100%). (C) Strength of the U46619-induced vasconstriction, referenced to the effect of the second U46619 application (= 100%). Data are derived from n = 5 isolated lung preparations each. * Significant differences compared to control experiments with application of the solvent only (p < 0.05).

In experiments with repetitive hypoxic ventilation maneuvers, normoxic PAP, assessed prior to each repetitive hypoxic challenge, was dose-dependently increased by OAG (Figure 2A). In parallel with this increase, the strength of HPV was diminished (Figure 2B). This effect was specific for HPV, since vasoconstriction induced by the thromboxane mimetic U46619 was not suppressed, but was rather increased by OAG (Figure 2C).

PAP measured after the initial steady state period, prior to the first vasoconstrictor provocation was 9.9 ± 0.1 mmHg (n = 20), and was not different between experiments with OAG application and solvent alone. Absolute ΔPAP values for the strength of HPV and U46619-induced vasoconstrictions were calculated at 1.6 ± 0.2 mmHg (n = 10) and 2.0 ± 0.2 mmHg (n = 10).

### The effect of the DAG kinase inhibitor R59949 on acute hypoxia-induced vasoconstriction

**Figure 3:**
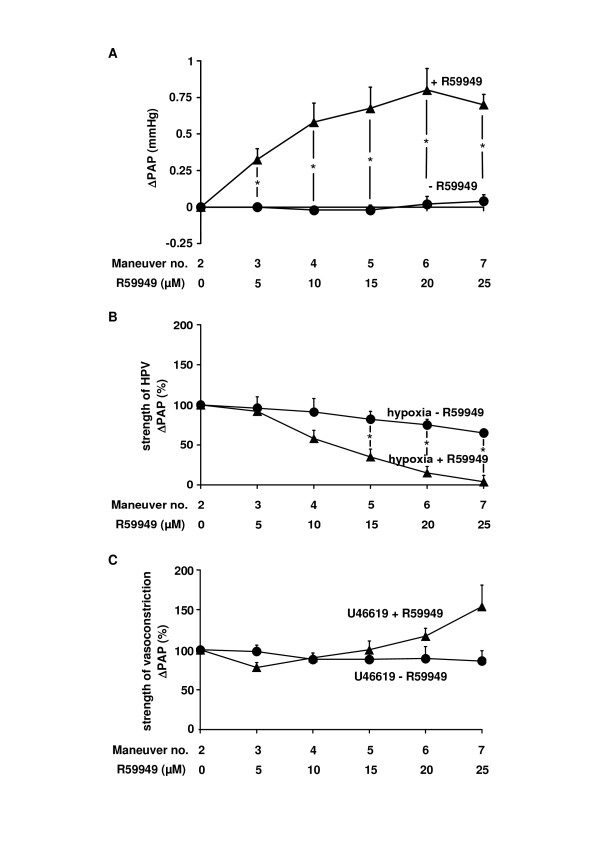
**Diacylglycerol kinase inhibitor R59949 diminished HPV specifically**. The effect of the diacylglycerol kinase inhibitor R59949 as well as its solvent on the normoxic vascular tone, the strength of HPV, and the strength of U46619-induced vasoconstriction was investigated in isolated wild-type mouse lungs. (A) Increase in normoxic pulmonary arterial pressure (ΔPAP), assessed directly before each hypoxic ventilation maneuver. (B) Strength of HPV referenced to the effect of the second hypoxic ventilation maneuver (= 100%). Absolute values of HPV for the second hypoxia maneuver prior to R59949 application were 0.7 ± 0.1 (n = 5). (C) Strength of the U46619-induced vasoconstriction, referenced to the effect of the second U46619 application (= 100%). Data are derived from n = 5 isolated lung preparations each. * Significant differences compared to control experiments with application of the solvent only (p < 0.05).

As DAG is degraded by DAG kinases [22,26], the effect of the DAG kinase inhibitor R59949 on normoxic PAP, the strength of HPV, as well as U46619-induced vasoconstrictions was investigated. As seen for OAG, the DAG kinase inhibitor dose-dependently increased normoxic PAP (Figure 3A). In parallel, the strength of HPV was reduced by R59949 and was completely abolished at 25 μM (Figure 3B). The inhibitory effect was specific for HPV, since R59949 caused no significant inhibition of U46619-induced vasoconstrictions, but amplification at higher concentrations (Figure 3C).

### The effect of the PLC inhibitor U73122 on acute hypoxia-induced vasoconstriction

**Figure 4:**
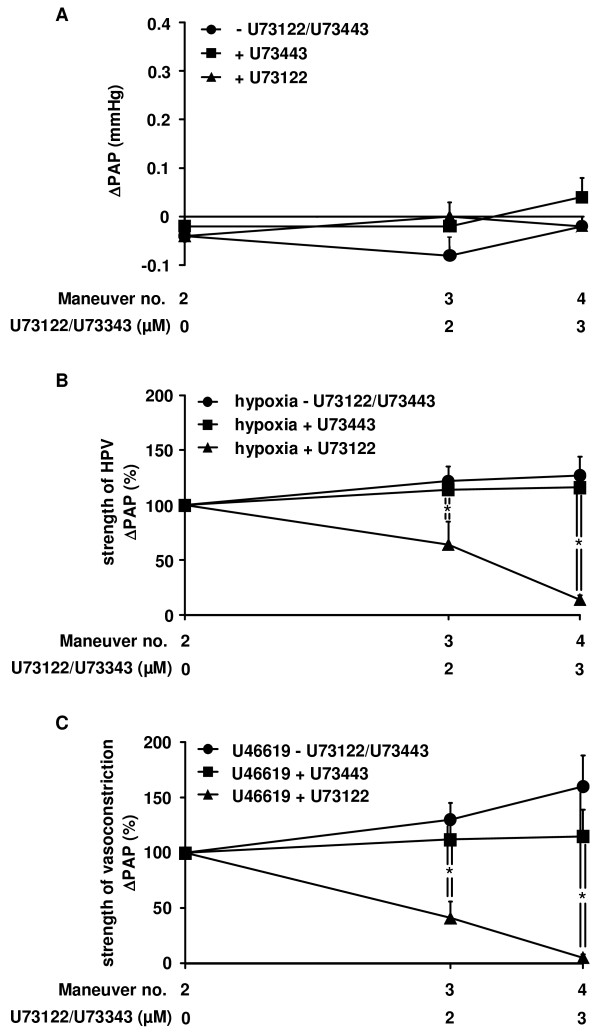
**Inhibitory effect of phospholipase C inhibitor U73122 on HPV and U46619-induced vasoconstriction**. The effect of the active (U73122) and inactive (U73343) form of phospholipase C inhibitor as well as their solvent on the normoxic vascular tone, the strength of HPV, and the strength of U46619-induced vasoconstriction was investigated in isolated wild-type mouse lungs. (A) Increase in normoxic pulmonary arterial pressure (ΔPAP), assessed directly before each hypoxic ventilation maneuver. (B) Strength of HPV referenced to the effect of the second hypoxic ventilation maneuver (= 100%). (C) Strength of U46619-induced vasoconstrictions, referenced to the effect of the second U46619 application (= 100%). Data are derived from n = 5 isolated lung preparations each. * Significant differences compared to control experiments with application of the solvent only (p < 0.05).

As PLC catalyzes the production of DAG [27], the impact of PLC on HPV was investigated by application of the PLC inhibitor U73122. As expected, U73122 did not significantly alter normoxic PAP. The same was true for control experiments with the inactive form of the PLC inhibitor (U73343) or the solvent of the PLC inhibitor (Figure 4A). In contrast, U73122 dose-dependently inhibited the strength of HPV (Figure 4B). However, U46619-induced vasoconstrictions were also diminished (Figure 4C). These effects were absent in experiments with the inactive compound, or the solvent of the PLC inhibitor applied alone.

### The effect of the DAG kinase inhibitor R59949 on sustained HPV

**Figure 5:**
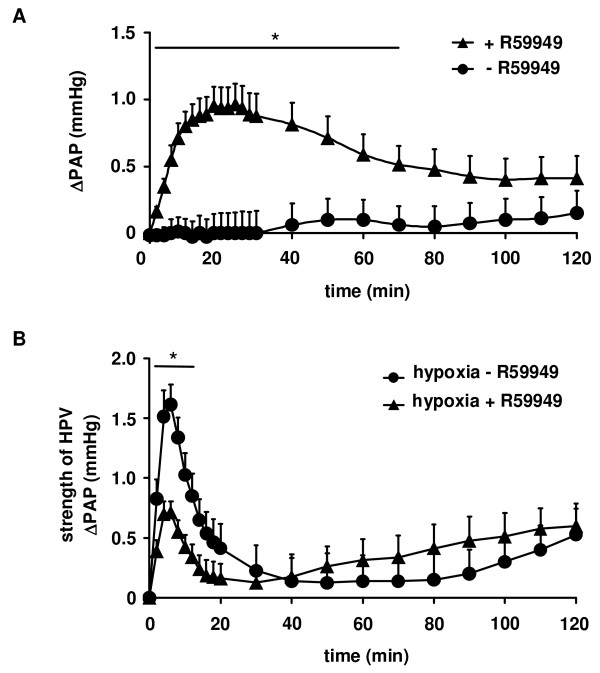
**Inhibition of acute but not sustained HPV by the diacylglycerol kinase inhibitor R59949**. The effect of the diacylglycerol kinase inhibitor R59949 as well as its solvent on normoxic vascular tone and sustained HPV was investigated for a period of 120 min. (A) Increase in normoxic pulmonary arterial pressure (ΔPAP) referenced to the value directly before R59949 application. For comparison changes in PAP are given for normoxic lungs in the absence of R59949. (B) Strength of sustained HPV in the presence and the absence of R59949. Data are derived from n = 8 isolated lung preparations each. * Significant differences compared to experiments in the absence of R59949 (p < 0.05).

A single application of 10 μM R59949, a concentration that inhibited acute HPV by approximately 50%, resulted in a transient vasoconstriction under normoxic ventilation. The maximum increase in PAP occurred after 18 ± 2 min (Figure 5a). Although this dosage still inhibited acute HPV when hypoxic ventilation was performed 120 min after a single application of R59949 (data not given), no sustained elevation of PAP was noted during continuous normoxic ventilation: significant differences in PAP in comparison to the solvent control could be detected for time points ≤ 70 min only (Figure 5a). If R59949 was applied prior to a 120 min period of hypoxic ventilation, only the acute but not the sustained phase of HPV was inhibited (Figure 5b).

## Discussion

The major finding of this study is that the initiation of acute HPV occurs via a DAG-mediated activation of TRPC6 in mice.

Regarding the vascular effects of alveolar hypoxia, it has previously been shown that these can be divided into three phases, one occurring within seconds, a second developing upon hypoxic ventilation of more than 20 min, and a third which includes a vascular remodeling process, permanently decreasing the area of the vascular lumen [12]. Although some heterogeneity as to the kinetics of the first two phases has been described [26,28-31], it was recently demonstrated that TRPC6 is essential for the acute phase, but not the sustained or chronic vascular effects in mice [9]. However, it has not yet been clarified which pathway controls TRPC6 in acute HPV. TRPC6 is a member of the DAG-sensitive TRPC3/6/7 subfamily, which has been shown to increase [Ca^2+^]_i _in a membrane-delimited fashion, independently of protein kinase C [32]. TRPC6 is insensitive to activation by inositol 1,4,5-trisphosphate at the cellular level [33]. As to the DAG-sensitive nature of TRPC6, it was hypothesized that an increase in DAG induces HPV via this channel. This hypothesis was derived from previous findings in isolated PASMC that 1) hypoxia causes an accumulation of DAG at the cell membrane and that 2) the DAG kinase inhibitor causes an increase in [Ca^2+^]_i _in these cells. In this study, it is documented for the first time in intact lungs that the membrane-permeable analog of DAG, OAG, induces a vasoconstriction in WT mice which is mediated via TRPC6. This can be concluded from the observation that the DAG-induced vasoconstriction was absent in TRPC6^-/- ^mice, although it has been previously demonstrated that TRPC6^-/- ^mice do not lack non-hypoxia-induced vasoconstrictor responses [9]. The latter finding excludes a general lack of vasocontractility in TRPC6-/- mice.

The central role of DAG in acute HPV was further supported by the effects of the DAG analog OAG, as well as the DAG kinase inhibitor and the phospholipase C inhibitor. Along these lines, it has been shown that DAG accumulation can be caused by inhibition of DAG kinases [34] but also by activation of PLC [35].

OAG did not only induce a vasoconstrictor response under normoxic conditions but also inhibited the subsequent acute HPV during hypoxia. Consistent with this idea, an increase in cellular DAG levels by the application of DAG kinase inhibitor [34] increased vasoconstriction, but decreased the subsequent acute HPV. Thus, both OAG and R59949 interfere with the same signal transduction cascade inducing acute HPV and therefore mimic this physiological response under normoxic conditions. The fact that low doses of OAG slightly increased the strength of HPV maybe is induced by the supplementary effect of the exogenous OAG in addition to the endogenous DAG produced by acute hypoxia. The inhibition of HPV caused by OAG or R59949 was specific for HPV as non-hypoxia-induced vasoconstriction induced by U46619 was not inhibited by either agent.

The notion that OAG-induced vasoconstrictions under normoxia were somewhat higher than the strength of HPV indicates, as expected, that DAG not only specifically mediates hypoxia-induced vasoconstrictions but also contributes to non-hypoxia-induced vasoconstrictions as previously shown for G protein coupled receptors [36].

In contrast to those agents which mimic acute HPV or increase DAG levels, the PLC inhibitor U73122 should decrease intracellular DAG production [37] and thus should suppress but not mimic HPV. As expected, while not mimicking acute HPV, U73122 suppressed HPV. It is unlikely that this effect is due to an unspecific inhibition of other signaling processes, because the inactive - but structurally analogous - compound U73343 had no effect on acute HPV. The fact, that U46619-induced vasoconstrictions were also suppressed by the PLC but not the DAG kinase inhibitor, supports the notion that the thromboxane mimetic-induced vasoconstriction is dependent on PLC without triggering the DAG-TRPC6-axis. In this regard it was shown that activation of thromboxane receptors can contribute to contraction of bovine pulmonary arteries by depletion of intracellular calcium stores and calcium entry via store-operated calcium channels [38]. Besides a role of store-operated calcium channels, U46619-induced contraction was also shown to be dependent at least in part on calcium entry through VOCC [35]. In this process, ROS-induced PKC zeta activation inhibited voltage-gated potassium (Kv) channel activity leading to membrane depolarization and activation of VOCC [36,37]. Moreover, PKC can generally also be activated by DAG [38]. Triggering different pathways, cell-compartmentalization, as well as some synergism of the above pathways may explain the increase of U46619-induced vasoconstrictions after application of the DAG analog OAG. Thus, the enhancement of U46619-induced vasoconstrictions, in contrast to the inhibition of HPV, further supports the specific role of DAG in acute HPV signaling. A compartmentalized effect of DAG regulation in HPV is further supported by the fact that the DAG kinase inhibitor R59449 did not amplify U46619-induced but selectively inhibited HPV.

The fact that the DAG kinase inhibitor R59449 inhibited only acute HPV but not sustained HPV and mimicked only acute but not sustained HPV during normoxic ventilation is well in line with our previous finding that DAG-regulated TRPC6 channels are essential for acute but not sustained HPV.

With regard to previous findings that acute HPV may be regulated by reactive oxygen species [39-43], we speculate that DAG levels activating TRPC6 can be increased by a redox-dependent mechanism. This can hypothetically be caused by a redox-dependent modulation of e.g. DAG kinase activity. In addition to TRPC6, it was shown that Kv channels are essential for acute HPV [44,45]. Although our current study did not investigate a possible effect of the DAG-TRPC6-axis on the closure of Kv channels in HPV, these two systems may be linked via modulation of the cellular sodium concentration as previously suggested [46]. This concept would be in line with the lack of acute HPV recently described in malonyl-CoA decarboxylase deficient mice, which lack hypoxic mitochondrial ROS signaling and Kv channel inhibition [40].

The specific role of DAG in mediating acute HPV shown in our isolated lung experiments (where the endothelium is present) and in our previous study [9] in isolated PASMC (where the increase in [Ca^2+^]_i _was used as a readout for HPV) indicates that the oxygen sensing process underlying HPV resides in the PASMC. This is well in line with numerous previous findings [47-50] and the notion expressed by others that acute HPV (in contrast to sustained) is independent from the endothelium [13,15,17,51]. This, however, does not exclude an important role of the endothelium in modulating HPV, though not contributing to oxygen sensing. This interpretation is well in line with the finding by us and others that isolated PASMC need to be "primed" by e.g. endothelin-1 as a prerequisite for functional oxygen sensing [9,52-54] and allows the suggestion that a basic stimulation of DAG production by G-protein coupled receptors is a prerequisite for oxygen sensing and signal transduction of acute HPV, occurring cell compartment-specific by DAG kinase inhibition.

## Conclusions

To summarize, DAG was identified as an important mediator in the signaling pathway underlying acute HPV. Moreover, these data indicate that DAG activates TRPC6 as an essential step in the mechanism of acute HPV.

## Competing interests

The authors declare that they have no competing interests.

## Authors' contributions

BF participated in the design, analysis and interpretation of experiments, performed statistical analysis, and designed and drafted the manuscript together with NW and AD. MR performed most of the experiments and has made substantial contributions to analysis and interpretation and statistical analysis of the data. HAG, RTS, WS, and FG participated in the design of the experiments and in the interpretation of the results. TG and AD supplied and/or generated the TRPC6^-/- ^mice and developed the hypothesis together with NW and BF. Their contribution was critical for intellectual content and designed portions of the experiments. NW designed and supervised the study, interpreted the data and drafted the manuscript together with BF and AD. All authors read and approved the final manuscript.
